# A rare recurrence of bilateral breast cancer in the esophagus coincidentally associated with primary gastric cancer: a case report

**DOI:** 10.1186/1752-1947-8-58

**Published:** 2014-02-18

**Authors:** Shinichiro Kashiwagi, Naoyoshi Onoda, Yuka Asano, Satoru Noda, Hidemi Kawajiri, Tsutomu Takashima, Tetsuro Ishikawa, Kosei Hirakawa

**Affiliations:** 1 Department of Surgical Oncology, Osaka City University Graduate School of Medicine, 1-4-3 Asahi-machi, Abeno-ku, Osaka, Japan

## Abstract

**Introduction:**

Cases of esophageal metastasis of breast cancer are extremely rare. We present the case of a patient who developed recurrence as esophageal metastasis following treatment of bilateral breast cancer. Early-stage gastric cancer was also found coincidentally.

**Case presentation:**

An 86-year-old Japanese female patient with a history of bilateral breast cancer was found to have a gastric mass on a medical examination. At 72 years of age, she had undergone a total mastectomy with level II axillary lymph node dissection (pT3N0M0 stage II). Left breast cancer was found at the age of 79. A total mastectomy was performed with level II axillary lymph node dissection (pT1N0M0 stage I). At the time of her current admission, our patient complained of dysphagia. A repeat gastrofiberscopy revealed a submucosal lesion in her middle esophagus, located 27cm distal to her incisors, as well as a known type I tumor of the gastric cardia. Computed tomography showed a mass lesion in her middle esophagus that had grown extraluminally and infiltrated the tracheal bifurcation and her left primary bronchus. A boring biopsy of the esophageal lesion was performed under ultrasonic monitoring, and a pathological diagnosis of poorly differentiated adenocarcinoma of the esophagus was obtained. The biopsy of the cardiac lesion revealed moderately differentiated adenocarcinoma of the stomach. The expression status of her hormone receptors indicated that the esophageal lesion reflected metastatic recurrence of her breast cancer with coincidental primary gastric cancer (cT1N0M0 stage IA).

**Conclusions:**

Esophageal metastasis of breast cancer is extremely rare. An individualized treatment plan combining multimodal approaches should therefore be devised according to the patient’s status.

## Introduction

Distant metastasis from primary breast cancer often occurs in the bones, lungs and liver. There are a few reports of metastasis to the gastrointestinal trunk [1], but cases of esophageal metastasis are extremely rare [2,3]. We present the case of a patient who developed recurrence as esophageal metastasis following treatment of bilateral breast cancer. Early-stage gastric cancer was also found coincidentally. To the best of our knowledge, there have been no previous reports of such a case. We present this case with a discussion of the diagnosis and treatment strategies.

## Case presentation

An 86-year-old Japanese female patient with a history of bilateral breast cancer was found to have a gastric mass on a medical examination. She was referred to our hospital for a workup and treatment. She had previously undergone surgery for right breast cancer at 72 years of age and subsequently underwent total mastectomy with level II axillary lymph node dissection. The pathological examination at that time revealed invasive ductal carcinoma with positive lymphovascular invasion (pT3N0M0 stage II). The tumor was positive for both estrogen receptor (ER) and progesterone receptor (PR), and did not overexpress human epidermal growth factor receptor 2 (HER2). A total of 50Gy of external beam irradiation therapy was administered to her residual mammary gland, followed by endocrine therapy with 20mg of daily tamoxifen administration for five years. She was found to have left breast cancer at the age of 79 years. A total mastectomy was performed with level II axillary lymph node dissection, and invasive ductal carcinoma with positive lymphovascular invasion (pT1N0M0 Stage I) was diagnosed. As before, the tumor was positive for ER and PR with no HER2 overexpression. She received extended adjuvant endocrine therapy of 1mg per day of anastrozole for five years.

**Figure 1:**
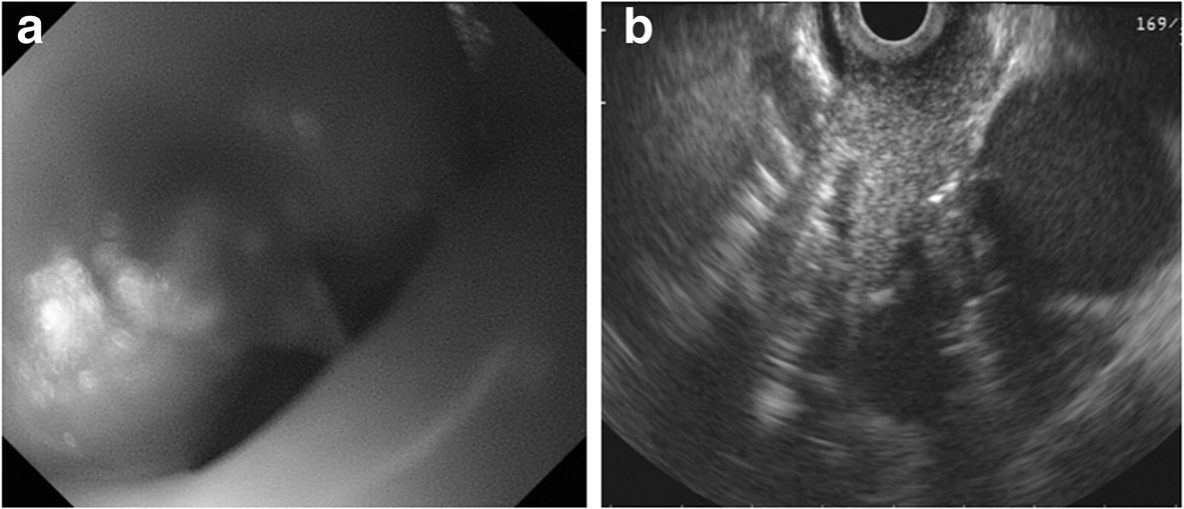
**Gastrofiberscopy and fine-needle aspiration cytology. (a)** Gastrofiberscopy revealed a submucosal lesion of the middle esophagus. **(b)** Fine-needle aspiration cytology and a boring biopsy of the esophageal lesion were obtained under ultrasonic monitoring.

**Figure 2:**
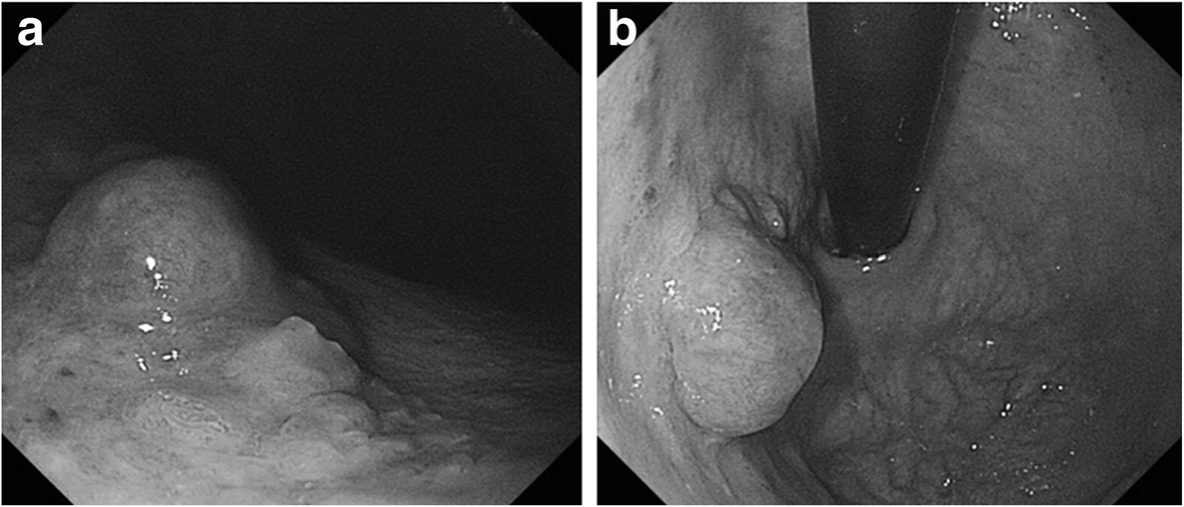
Gastrofiberscopy revealed a type I tumor (semipedunculated type) of the gastric cardia (a, b).

**Figure 3:**
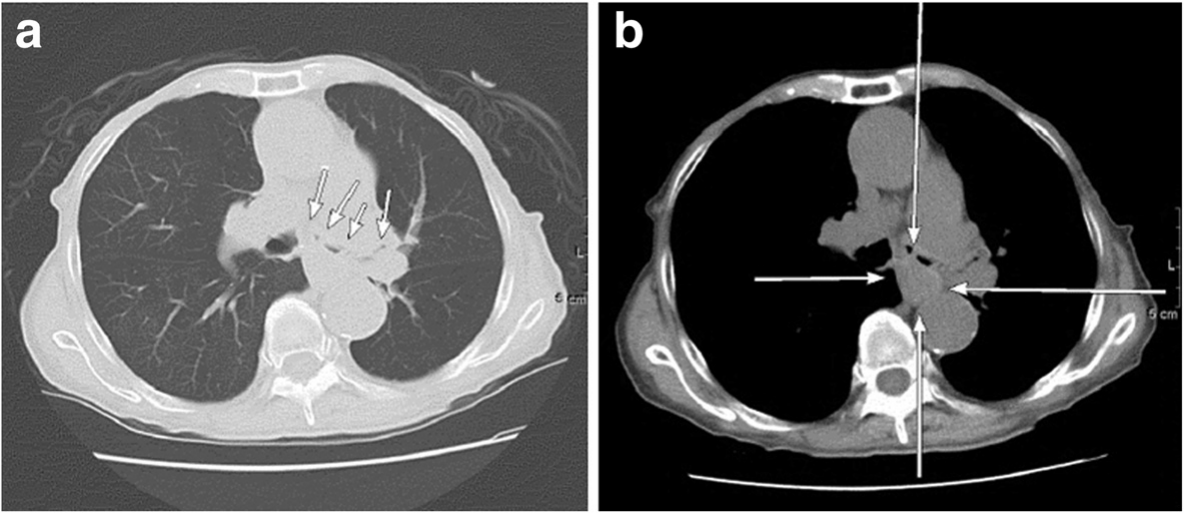
Mediastinal computed tomography showed a mass lesion on the middle esophagus that had grown extraluminally and had infiltrated the tracheal bifurcation and the left primary bronchus (a, b).

**Figure 4:**
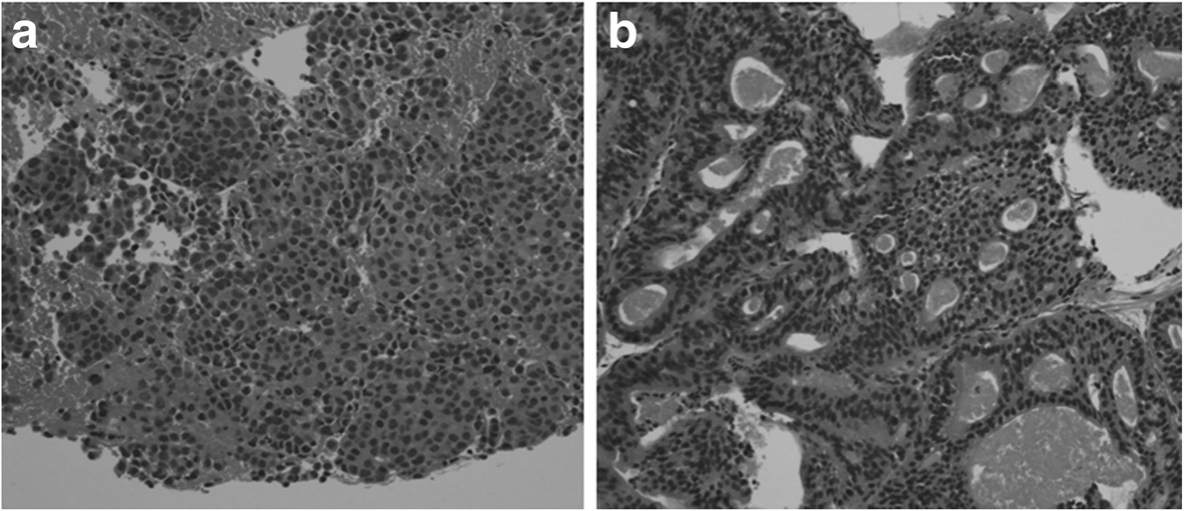
**Histological findings of the biopsy. (a)** Poorly differentiated adenocarcinoma of the esophagus. **(b)** Adenocarcinoma of the stomach.

On her current admission, our patient complained of dysphagia. Repeat gastrofiberscopy revealed a submucosal lesion in her middle esophagus located 27cm distal to her incisors (Figure 1a), as well as the known type I tumor (semipedunculated type) of her gastric cardia (Figure 2). Biochemistry and blood testing did not reveal any abnormal values. Her level of squamous cell carcinoma antigen was elevated (5.4ng/mL); however, the values of the other markers (carcinoembryonic antigen, carbohydrate antigen 15–3, National Cancer Center-ST 439, cytokeratin 19 fragment) were within the normal ranges. Mediastinal computed tomography showed a mass lesion in her middle esophagus that had grown extraluminally and infiltrated the tracheal bifurcation and her left primary bronchus (Figure 3). There was no hepatic or pulmonary metastasis. Fine-needle aspiration cytology and a boring biopsy of the esophageal lesion were performed under ultrasonic monitoring (Figure 1b), and a pathological diagnosis of poorly differentiated adenocarcinoma of the esophagus was obtained with positive ER staining on immunohistochemistry (Figure 4a). A biopsy of the cardiac lesion revealed moderately differentiated adenocarcinoma of the stomach (Figure 4b). Neither hormone receptors nor HER2 were expressed in the gastric lesion. The expression status of the hormone receptors indicated that the esophageal lesion reflected metastatic recurrence of her breast cancer with coincidental primary gastric cancer (cT1N0M0 stage IA).

**Figure 5:**
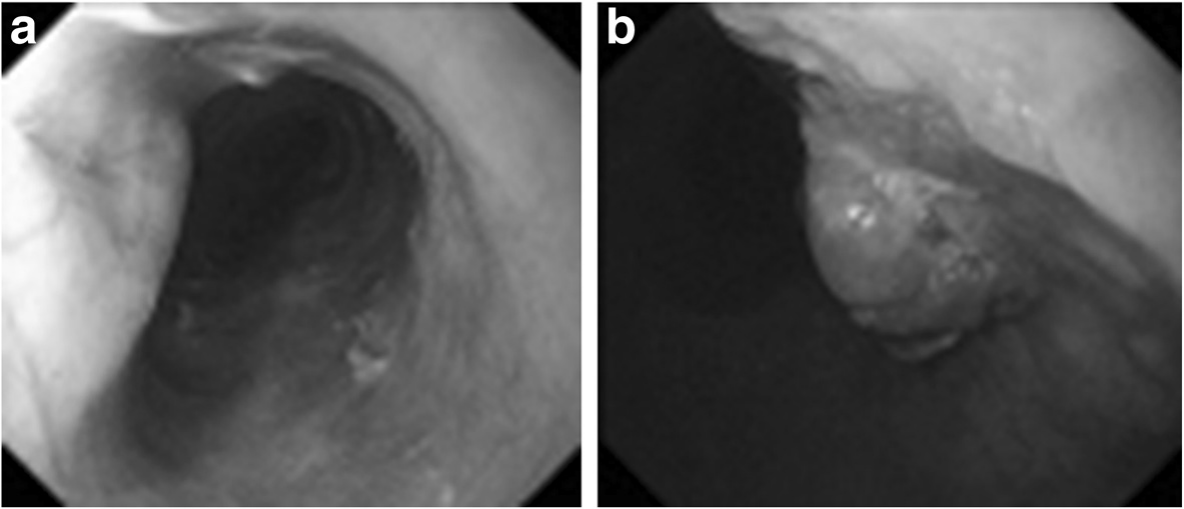
**Gastrofiberscopy after radiotherapy. (a)** The esophageal tumor was remarkably reduced in size. **(b)** The cardiac tumor showed no remarkable change.

Although our patient’s dysphagia progressed, neither intensive chemotherapy nor highly invasive surgical therapy were considered to be indicated due to her general condition and age. The esophageal lesion was treated with 2Gy per day of external beam irradiation therapy for 30 days (for a total of 60Gy). It remarkably reduced in size after radiotherapy (Figure 5a), allowing oral inoculation. The cardiac tumor exhibited no remarkable changes (Figure 5b). Endoscopic submucosal dissection was considered for the gastric tumor. Unfortunately, endoscopic ultrasonography (EUS) demonstrated that the tumor had invaded her submucosal layer and it was judged to be inoperable due to the risk of bleeding and perforation. The administration of chemotherapy and endocrine therapy for her breast cancer was prioritized because the esophageal lesion of the breast cancer was much more advanced and critical for our patient’s status than the gastric cancer. She received metronomic medication with daily oral administration of tegafur-uracil (400mg per kilogram body weight), cyclophosphamide (100mg/day) and medroxyprogesterone acetate (800mg/day) based on her age and therapeutic history, as well as the biological subtype of the tumor and presence of the non-treated gastric cancer. She has since maintained a good performance status on outpatient treatment with chemo-endocrine therapy, and no disease progression had been noted to date, six months after the radiation therapy.

## Discussion

Gastrointestinal metastasis of breast cancer is rare, with an incidence of 4.3% to 14.4% [1]. Although gastric metastasis of breast cancer is sometimes reported [1], metastasis to the esophagus occurs extremely rarely. According to Borst and Ingold, who observed a total of 2,246 patients with breast cancer over 18 years, the incidence of esophageal metastasis was only 0.4% [4]. There are very few case reports of such metastasis [5-19] (Table 1); based on a search of PubMed, only 20 cases have been reported. We found no reports describing a case of gastric metastasis of breast cancer accompanied by gastric cancer or occurring after treatment for bilateral breast cancer.

**Table 1:** Reported cases of esophageal metastasis from breast cancer

**Author**	**Year**	**Age**	**Location**	**Latent interval (years)**	**Treatment**	**Survival**
Haim *et al.*[5]	1989	50	Mt	12	D/HT/RT	1 year/AOD
Issacs *et al.*[6]	1989	60	Mt, Lt	5	D/HT	-/-
Shimada *et al.*[7]	1989	55	Lt	9	HT/OP	5 years/AOD
Herrera [8]	1992	78	EGJ	21	HT/OP	Postop/DOD
Labenz *et al.*[9]	1993	61	Mt	-	S	0.4 years/AOD
Hastier *et al.*[10]	1994	42	Lt	9		-/DOD
Varanasi *et al.*[11]	1995	63	Mt	8	D/OP	-/-
		64	Mt	22	D/OP/RT	Postop/DOD
		78	Mt	8	D/OP	Postop/DOD
		67	Mt, Lt	22	D/HT/RT	4 years/DOD
Mizobuchi *et al.*[12]	1997	56	Lt	7	CT/HT/OP	4 years/DOD
Fujii *et al.*[13]	1997	68	Mt	15	HT/OP	1 year/AOD
Wu *et al.*[14]	1998	83	Mt	13	D/HT	-/-
Simchuk and Low [2]	2001	73	Lt	9	D	1 year/DOD
		41	Mt	4	CT/D	0.1 years/DOD
		77	Mt	3.5	D	1.5 years/AOD
		74	Mt	11	D/S	0.4 years/DOD
Erman *et al.*[15]	2002	55	Mt	11	CT/HT/RT	3 years/AOD
Sunada *et al.*[16]	2005	68	Mt	24	D/HT	0.7 years/AOD
Koike *et al.*[17]	2005	72	Mt	23	OP	1 year/AOD
		52	Lt	10	D/HT/S	-/-
Anaya *et al.*[18]	2006	67	EGJ	19	HT/OP	0.5 years/AOD
Wada *et al.*[19]	2009	70	Mt	16	HT/RT	2.5 years/AOD
Present case	2011	86	Mt	7	HT/RT	0.5 years/AOD

AOD, alive of disease; CT, chemotherapy; D, dilatation; DOD, dead of disease; EGJ, esophagogastric junction; HT, hormonal therapy; Lt, lower thoracic esophagus; Mt, middle thoracic esophagus; OP, operation; RT, radiation therapy; S, stenting.

Esophageal metastasis of breast cancer is usually detected at an average of approximately seven years (range 3.5 to 24 years) after breast resection [20]. Symptoms include dysphagia, hoarseness and weight loss; however, such symptoms are observed only in approximately 30% of patients [21]. Esophageal metastasis of breast cancer causes compression or stenosis of the esophagus due to metastasis within the esophageal wall or to the mediastinal lymph nodes near the esophagus. Therefore, the mucosal surface is usually not involved. These features were also found in our patient. Esophagography and endoscopy are the diagnostic tools. The findings of these tests are characterized by regular stenosis of the esophagus as well as a lack of abnormalities of the mucosal surface. Biopsies often do not reveal malignant findings. Therefore, submucosal boring biopsies and aspiration cytology are recommended for biopsy-based diagnosis. Our patient was given a diagnosis based on successful EUS-guided fine-needle aspiration cytology and a boring biopsy, an alternative method for obtaining submucosal tumor tissues. Most esophageal metastases originating from breast cancer exhibit submucosal lesions [5-19]. Therefore, patients presenting with dysphagia should not be assumed to have benign stricture involvement even if a mucosal biopsy does not demonstrate malignancy. As a result, the indications for EUS are useful in such situations, as observed in our case.

The prognosis of metastatic breast cancer to the esophagus is poor, and it is reasonable to regard the disease as a highly advanced breast cancer [2,20]. Therefore, the therapeutic strategy should always be individually planned. According to a literature review, surgical therapy has been performed in nine patients, four of whom survived for more than one year [7,8,11-13,17,18]. This result justifies the use of surgical treatment when deemed possible. By contrast, three patients died postoperatively, suggesting the highly invasive nature of surgical therapy when adapted to esophageal lesions [22]. The remaining patients were treated with dilatation, stenting and/or radiation in 13, three and six cases, respectively [5-19]. Only short-term follow-up results are available for patients who underwent dilatation and stenting (9). Radiation was administered in six cases [5,11,15,19], with a relatively good prognosis of more than one year reported for every patient. All five patients who survived for more than two years received hormone therapy in combination with local treatment with either surgery or radiation. These results indicate that patients exhibiting a response to hormonal therapy, as in our case, may be expected to demonstrate a more favorable prognosis than patients with hormone receptor-negatives tumors. According to our review, as many as 13 of 24 patients with metastatic breast cancer to the esophagus showed positive results for ER and/or PR [5-8,11,17-19]. Therefore, it is thought that active treatment should be provided based on the intrinsic subtype. It is important to administer appropriate multimodal therapy with topical treatment for esophageal lesions (for example, radiotherapy, esophageal bougienage and esophagectomy) [7,20] and systemic treatment for distant metastasis (for example, chemotherapy and endocrine therapy).

The administration of individualized treatment according to the intrinsic subtype is recommended in patients with breast cancer. Endocrine therapy was selected in our case because our patient’s breast cancer was classified as luminal A type (ER-positive, HER2-negative, Ki67 <14%). Toremifene, an aromatase inhibitor, was chosen for endocrine therapy because tamoxifen had been used for five years for the right breast cancer that developed at 72 years of age, and anastrozole for the same duration for the left breast cancer, possibly the lesion responsible for the present metastasis, that developed at 79 years of age. The primary tumors of both her breasts and the metastatic lesion demonstrated the same biological nature (ER-positive and HER2-negative). However, untreated early gastric cancer was also coincidentally found in our patient. Therefore, combination therapy was administered with tegafur-uracil, cyclophosphamide and medroxyprogesterone acetate. This combination therapy is reported to be useful for elderly patients [23].

## Conclusions

Esophageal metastasis of breast cancer is extremely rare. An individualized treatment plan combining multimodal approaches should therefore be devised according to the patient’s status.

## Consent

Written informed consent was obtained from the patient for publication of this case report and accompanying images. A copy of the written consent is available for review by the Editor-in-Chief of this journal.

## Abbreviations

ER: estrogen receptor; HER2: human epidermal growth factor receptor 2; PR: progesterone receptor.

## Competing interests

The authors declare that they have no competing interests.

## Authors’ contributions

SK, HK and TT performed clinical work. SK, YA, SN, NO, TI and KH carried out laboratory work. All authors contributed to writing the article. All authors read and approved the final manuscript.

## References

[B1] Davis HL Murray RK Korbiz BC Breast carcinoma metastatic to the stomach Am J Dig Dis New Ser 1968 13 868 873 10.1007/BF02237571 5303029

[B2] Simchuk EJ Low DE Direct esophageal metastasis from a distant primary tumor is a subclinical process: a review of six cases Dis Esophagus 2001 14 247 250 10.1046/j.1442-2050.2001.00195.x 11869331

[B3] Kamby C Vejborg I Kristensen B Olsen LO Mouridsen HT Metastatic patterns in recurrent breast cancer: special reference to intrathoracic recurrences Cancer 1988 62 2226 2233 10.1002/1097-0142(19881115)62:10<2226::AID-CNCR2820621026>3.0.CO;2-D 3179937

[B4] Borst MJ Ingold JA Metastatic patterns of invasive lobular versus invasive ductal carcinoma of the breast Surgery 1993 114 637 641 8211676

[B5] Haim N Krugliak P Cohen Y Schwartzman Z Benharroch D Esophageal metastasis from breast carcinoma associated with pseudoepitheliomatous hyperplasia: an unusual endoscopic diagnosis J Surg Oncol 1989 41 278 281 10.1002/jso.2930410416 2547117

[B6] Isaacs P MacGillivray N Springett P Late recurrence of breast cancer presenting with esophageal dysmotility J Clin Gastroenterol 1989 11 588 590 2794442

[B7] Shimada Y Imamura M Tobe T Successful esophagectomy for metastatic carcinoma of the esophagus from breast cancer: a case report Jpn J Surg 1989 19 82 85 10.1007/BF02471573 2471861

[B8] Herrera JL Benign and metastatic tumors of the esophagus Gastroenterol Clin North Am 1991 20 775 789 1787013

[B9] Labenz J Madeya S Boerch G Post-mastectomy dysphagia: successful treatment with an esophageal self-expanding metallic stent Gastrointest Endosc 1993 39 599 10.1016/s0016-5107(93)70190-x 7689994

[B10] Hastier P Francois E Delmont JP Harris AG Barthel HR Namer M Esophageal metastasis from breast cancer detected by hematemesis Am J Gastroenterol 1994 89 289 290 8304327

[B11] Varanasi RV Saltzman JR Krims P Crimaldi A Colby J Breast carcinoma metastatic to the esophagus: clinicopathological and management features of four cases, and literature review Am J Gasteroenterol 1995 90 1945 1499 7661177

[B12] Mizobuchi S Tachimori Y Kato H Watanabe H Nakanishi Y Ochiai A Metastatic esophageal tumors from distant primary lesions: report of three esophagectomies and study of 1,835 autopsy cases Jpn J Clin Oncol 1997 27 410 414 10.1093/jjco/27.6.410 9438004

[B13] Fujii K Nakanishi Y Ochiai A Tsuda H Yamaguchi H Tachimori Y Kato H Watanabe H Shimoda T Solitary esophageal metastasis of breast cancer with 15 year’ latency: a case report and review of the literature Pathol Int 1997 47 614 617 10.1111/j.1440-1827.1997.tb04550.x 9311012

[B14] Wu CM Hruban RH Fishman EK Breast carcinoma metastatic to the esophagus Clin Imaging 1998 22 343 345 10.1016/S0899-7071(98)00027-8 9755397

[B15] Erman M Karaoğlu A Oksüzoğlu B Aydingöz U Ayhan A Güler N Solitary esophageal metastasis of breast cancer after 11 years Med Oncol 2002 19 171 175 10.1385/MO:19:3:171 12482128

[B16] Sunada F Yamamoto H Kita H Hanatsuka K Ajibe H Masuda M Hirasawa T Osawa H Sato K Hozumi Y Sugano K A case of esophageal stricture due to metastatic breast cancer diagnosed by endoscopic mucosal resection Jpn J Clin Oncol 2005 35 483 486 10.1093/jjco/hyi123 16006575

[B17] Koike M Akiyama S Kodera Y Nakao A Breast carcinoma metastasis to the esophagus: report of two cases Hepatogastroenterology 2005 52 1116 1118 16001642

[B18] Anaya DA Yu M Karmy-Jones R Esophageal perforation in a patient with metastatic breast cancer to esophagus Ann Thorac Surg 2006 81 1136 1138 10.1016/j.athoracsur.2005.01.052 16488749

[B19] Wada Y Harada N Ohara K Kawata H Iwasaki H Kawamura Y Gomi T Ohtoshi M Nakashima Y Esophageal metastasis of breast carcinoma Breast Cancer 2009 16 151 156 10.1007/s12282-008-0068-6 18762863

[B20] Anderson MF Harell GS Secondary esophageal tumors Am J Roentogenol 1980 68 149 153 10.2214/ajr.135.6.1243 6779532

[B21] Goldberg RI Rams H Stone B Barkin JS Dysphagia as the presenting symptom of recurrent breast carcinoma Cancer 1987 60 1085 1088 10.1002/1097-0142(19870901)60:5<1085::AID-CNCR2820600527>3.0.CO;2-8 3300948

[B22] Orringer MB Skinner DB Unusual presentations primary and secondary esophageal malignancies Ann Thorac Surg 1971 11 305 314 10.1016/S0003-4975(10)65453-7 5548975

[B23] Ogawa Y Ishikawa T Chung SH Ikeda K Takashima T Onoda N Nakata B Nishiguchi Y Hirakawa K Oral UFT and cyclophosphamide combination chemotherapy for metastatic breast cancer Anticancer Res 2003 23 3453 3457 12926089

